# XIAP induces inflammation and promotes colorectal cancer progression

**DOI:** 10.1038/s41419-026-09391-9

**Published:** 2026-11-02

**Authors:** Lars M. Schiffmann, Mila Daoud, Claudia Maria Wunderlich, Fabian Schorn, Johanna Stachelscheid, Pia Nora Broxtermann, Marie-Christine Albert, M. Siebenbürger, Heike Göbel, Ulrike Göbel, Ali T. Abdallah, J. Paul Werthenbach, Jens Michael Seeger, Naita M. Wirsik, Karl Knipper, Su Ir Lyu, Thomas Schmidt, Nikolai Schleußner, Uraz Yasar, Christian Goddemeier, Stephanie T. Jünger, Hans A. Schlößer, Georg Dieplinger, Thomas Zander, Hakan Alakus, Hans F. Fuchs, Florian Gebauer, Christiane J. Bruns, Domagoj Vucic, Alexander Quaas, Melanie Fritsch, Frank Thomas Wunderlich, Hamid Kashkar

**Affiliations:** 1 University of Cologne, Faculty of Medicine and University Hospital of Cologne, Department of General, Visceral, Thoracic and Transplantation Surgery, Cologne, Germany; 2 University of Cologne, Faculty of Medicine and University Hospital of Cologne, Institute for Molecular Immunology, Cologne, Germany; 3 Max Planck Institute for Metabolism Research, Department of Obesity and Cancer, Cologne, Germany; 4 University of Cologne, Faculty of Medicine and University Hospital of Cologne, Institute of Pathology, Cologne, Germany; 5 University of Cologne, Cologne Excellence Cluster on Cellular Stress Responses in Aging-Associated Diseases (CECAD), Cologne, Germany; 6 University Hospital Heidelberg, University Heidelberg, Department of General, Visceral and Transplantation Surgery, Heidelberg, Germany; 7 University of Cologne, Faculty of Medicine and University Hospital of Cologne, Department of Neurosurgery, Cologne, Germany; 8 University of Cologne, Faculty of Medicine and University Hospital Cologne, Department I of Internal Medicine, Center of Integrated Oncology Aachen Bonn Cologne Duesseldorf, Cologne, Germany; 9 Immunology and Early Discovery Biochemistry, Genentech, South San Francisco, CA USA; 10 University of Cologne, Faculty of Medicine and University Hospital of Cologne, Center for Molecular Medicine Cologne (CMMC), Cologne, Germany

**Keywords:** Colon cancer, Oncogenesis

## Abstract

Colorectal cancer (CRC) is one of the most diagnosed malignancies worldwide, with chronic inflammation playing a critical role in its pathogenesis and progression. Surgery represents the primary treatment option, though some patients suffer from relapse, leading to considerable morbidity and mortality. A better molecular understanding of CRC pathogenesis is therefore essential for identifying patients at increased risk of recurrence and improving clinical management. Here we identified the X-linked inhibitor of apoptosis protein (XIAP) as an independent predictor of overall survival in primary resected CRC patients. To model CRC patients with low *versus* high XIAP expression, we established conditional mouse lines for intestinal epithelial cell (IECs)-specific *Xiap* knock-out and *XIAP* knock-in, and employed a colitis-associated murine model for CRC. We found that XIAP promotes inflammatory signalling and exacerbates colitis-driven colorectal tumorigenesis by shaping a distinct tumor inflammatory microenvironment characterized by increased immune-cell infiltration, including neutrophils, and enhanced vascularisation. The pro-inflammatory role of XIAP was further validated in CRC patient-derived organoids and human CRC tumor specimens. Collectively, our findings identify XIAP as a potential biomarker of CRC-associated inflammation and a candidate marker for identifying patients at increased risk of disease recurrence. Moreover, these results provide a rationale for exploring XIAP-targeting strategies as a potential component of multimodal therapeutic approaches for CRC.

## Introduction

Colorectal cancer (CRC) ranks second on the list of leading causes of cancer-related deaths [1]. Over the last decades, tremendous efforts have been made for the early detection, operative techniques and multimodal treatment regimens that consistently prolonged patients’ survival [2–4]. Prognosis of CRC is highly dependent on the initial tumor stage with a 5-year overall survival for all stages of 63%. Patients with localised CRC have a prognosis with a 5-year survival of around 90% [5]. However, some of these patients with early-stage CRC suffer from relapse and progressive disease. Accordingly, additional clinical-pathological or molecular information to identify patients who are at risk of recurrence in early tumor stages would be of immense value. Notably, although several genetic aberrations have been associated with CRC, mutational make-up of tumor cells alone cannot be used as an accurate predictor for patient outcome. Instead, tumor microenvironment (TME) and in particular tumor-infiltrating immune cells are increasingly considered to dictate tumor progression making CRC a prototypical example of inflammation-driven cancer [6].

X-linked inhibitor of apoptosis protein (XIAP) is a RING E3 ubiquitin ligase, which was primarily shown to inhibit caspases and apoptosis [7, 8]. Additional data showed that XIAP also controls inflammatory cell death in response to TNF or by controlling inflammasome activation [9–12]. Independently, given its role in apoptosis and its frequently elevated expression in malignant cells, XIAP has garnered the most attention as a promising therapeutic target in cancer to overcome drug resistance [13–16]. While many studies indicated that XIAP is conducive for tumor progression, its anti-apoptotic function as a cancer driver has been a matter of controversy [14, 15, 17–19]. Independent of its anti-apoptotic activity, XIAP has long been known to induce cellular inflammatory signalling, motility, and plasticity by acting as a ubiquitin ligase (Ub-ligase) and activating NFκB and MAP kinase signalling [20–26]. Conventional and conditional *Xiap* inactivation in mice failed to demonstrate phenotypic alterations caused by increased apoptosis [27, 28]. XIAP-deficient mice, however, were shown to be susceptible to bacterial infection, identifying XIAP as an important mediator of inflammation and innate immunity [28–30]. Specifically, XIAP induces inflammation by ubiquitylation of the receptor-interacting protein kinase 2 (RIPK2) which leads to NFκB activity and inflammatory cytokine production [20, 23, 31–35]. The pro-inflammatory role of XIAP in cancer is, however, rarely studied. Besides extensive studies utilising *Xiap* gene knock-down or knock-out in cultured tumor cells [14, 36–40] experimental models addressing whether and how elevated XIAP expression serves as an oncogene or tumor promotor have been missing.

In the current study, we investigated the role of XIAP in CRC pathogenesis by analysing patients and utilising novel genetically engineered mice. We found that XIAP expression in primary resected human CRCs is an independent predictor of overall survival. By specifically ablating the *Xiap* gene or overexpressing XIAP in intestinal epithelial cells (IECs), we could show that XIAP promotes CRC progression in a colitis-driven tumor mouse model. Our data collectively indicated that XIAP fosters tumor growth by inducing cytokine/chemokine production, promoting tumor immune cell infiltration, and facilitating tumor angiogenesis. Based on these observations, we propose the use of XIAP-specific antagonists as potentially relevant complement for multimodal therapy regimens to treat CRC.

## Results

**Fig. 1:**
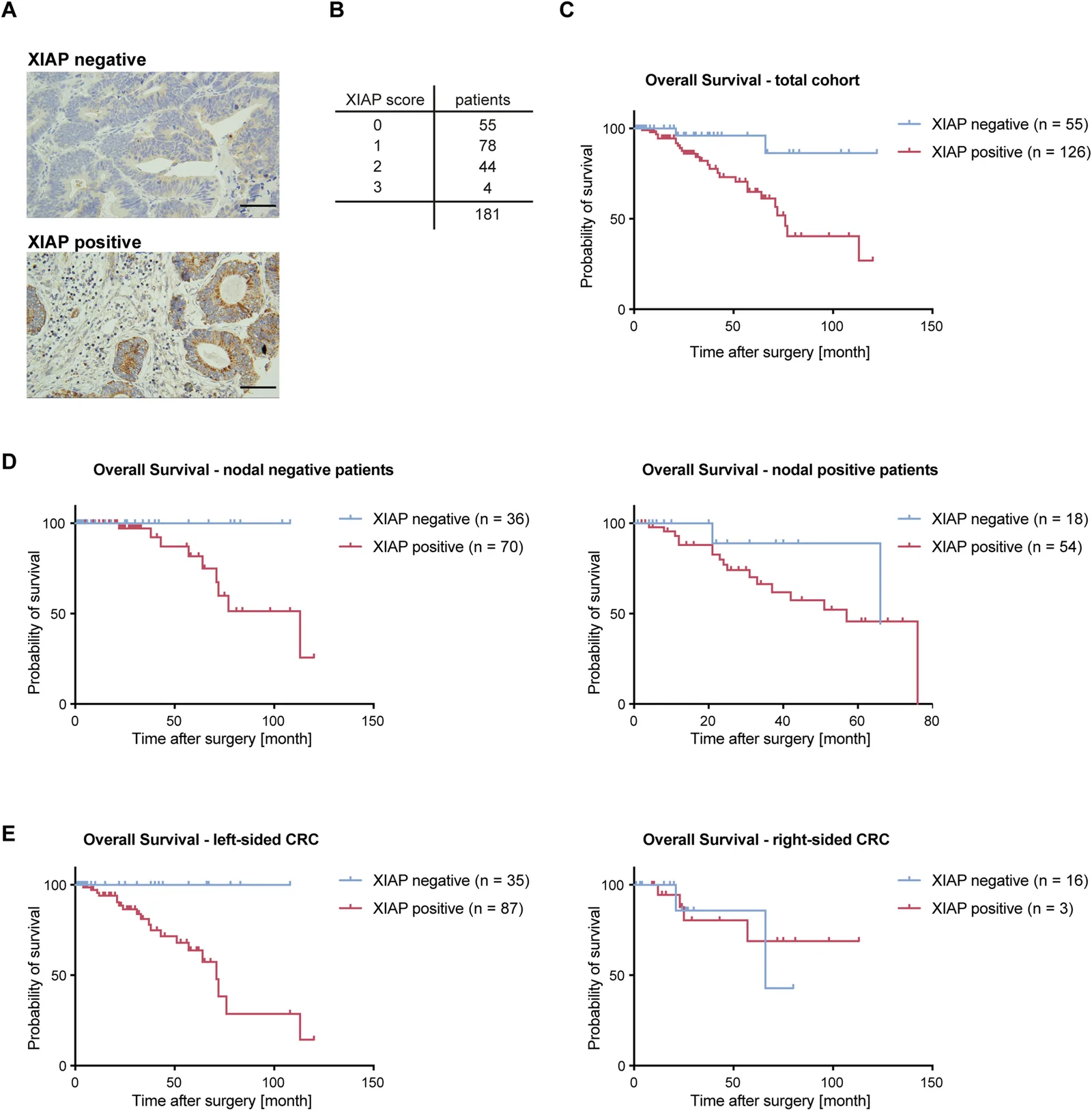
XIAP expression and its impact on overall survival of CRC patients. **A** Representative immunohistochemistry images of CRC sections depicting XIAP negative and XIAP positive tissue micro array spots. Bar indicates 50 µm. **B** Table showing the distribution of the immunohistopathological XIAP score 0-3 in primary CRC tumors. **C** Kaplan-Meier curve showing overall survival of patients with XIAP positive vs XIAP negative tumors, *p* = 0.007, log-rank test. **D** Kaplan-Meier curve showing overall survival of patients with XIAP positive vs XIAP negative tumors, stratified for their nodal status, *p* = 0.013 (left), *p* = 0.371 (right), log-rank test. **E** Kaplan-Meier curve showing overall survival of patients with XIAP positive vs XIAP negative tumors, stratified for the primary tumor location, *p* = 0.002 (left), *p* = 0.643 (right), log-rank test.

To examine the prognostic value of XIAP in patients with CRC, we analysed a cohort of patients that underwent surgery in curative intent for CRC. We first examined the XIAP protein expression in primary tumor samples by immunohistochemistry (Fig. 1A). XIAP expression was scored in four categories from 0 to 3, with 0 representing very weak or no XIAP staining and 3 a very strong staining (Fig. 1B). Our data showed that the overall survival gradually decreased when XIAP was expressed (Figure S1A). As XIAP scores 1 and 2 showed very similar Kaplan-Meier survival probabilities and score 3 represented a rather rare finding, we decided to stratify our findings in two main groups with the XIAP score 0 as XIAP negative and scores 1-3 as XIAP positive. Accordingly, XIAP-negative patients showed a strong survival benefit when compared to XIAP-positive patients (Fig. 1C). Notably, patients stratified in these two groups showed no statistical differences regarding the basic clinical characteristics (Figure S1B).

Multivariate analysis (Cox regression) identified XIAP as an independent factor predicting overall survival (Figure S1C). In nodal-negative patients, the overall survival of patients was impressively good when XIAP was not expressed (Fig. 1D). In contrast, XIAP expression seemed to be irrelevant in patients with nodal positivity. Further analysis after stratification of our patient collective in left-sided (distal colon) and right-sided colon (proximal colon) cancer revealed that XIAP expression was associated with low survival probability of patients with left-sided CRC (Fig. 1E), while its expression did not correlate with patients’ outcome affected by right-sided colon cancer.

**Fig. 2:**
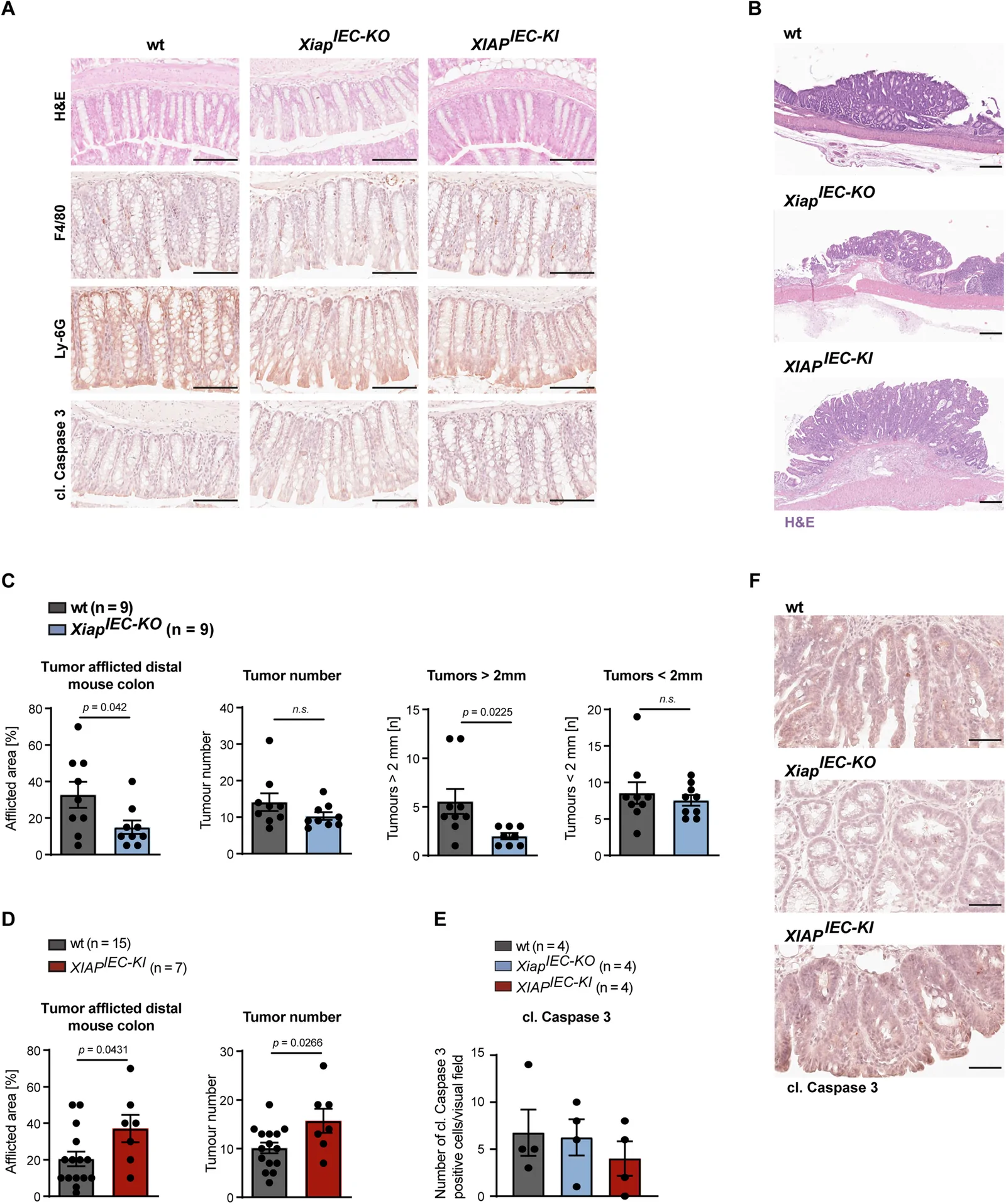
XIAP fosters colitis-driven colorectal tumor formation in mice without impacting on IEC apoptosis. **A** Representative images of colon sections of untreated wt (*n* = 3), *Xiap*^*IEC-KO*^ (*n* = 2) and *XIAP*^*IEC-KI*^ (*n* = 3) mice. Bars indicate 150 µm for H&E, and 100 µm for F4/80, Ly-6G, and cl. Caspase 3. **B** Representative images of haematoxylin/ eosin (H&E) stained sections of 2% AOM/DSS induced murine colorectal tumors of wt, *Xiap*^*IEC-KO*^ and *XIAP*^*IEC-KI*^ mice. Bar indicates 250 µm. **C** Percentage of tumor afflicted area, total tumor number, larger tumors and smaller tumors from these experiments comparing wt and *Xiap*^*IEC-KO*^ mice. Dots, individual mice. Lines, mean ± s.e.m. *P*-values by unpaired t-test. **D** Percentage of tumor afflicted area and total tumor number in wt and *XIAP*^*IEC-KI*^ mice. Dots, individual mice. Lines, mean ± s.e.m. *P*-values by unpaired t-test. **E** Quantification of cl. Caspase 3 positive cells per visual field of 2% AOM/DSS induced murine colorectal tumors of wt, *Xiap*^*IEC-KO*^ and *XIAP*^*IEC-KI*^ mice. Dots, individual mice. Lines, mean ± s.e.m. **F** Representative immunohistochemistry images of cl. Caspase-3 stainings of mouse adenomas of wt, *Xiap*^*IEC-KO*^ and *XIAP*^*IEC-KI*^ mice. Bar indicates 50 µm.

To experimentally examine the role of XIAP in CRC pathogenesis, we generated two different genetically engineered mouse lines enabling intestinal epithelial cell (IEC)-specific *Xiap* knock-out (KO) or *XIAP* knock-in (KI) (Figure S2A-B). To mimic XIAP-negative human CRC tumors, IEC-specific *Xiap*-KO was established by crossing *Xiap*^*fl/fl*^ [28] with *Villin*^*Cre*^ mice [41] (*Xiap*^*IEC-KO*^). *Xiap*^*IEC-KO*^ mice appeared normal (monitored for 6 months) and no obvious phenotypic alterations could be detected in the ileum (Figure S2C) or colon (Fig. 2A) of these mice. At ages of 8 weeks, mice were exposed to the azoxymethane/dextran sodium sulphate (AOM/DSS) protocol (Figure S2D) which induces a colitis-driven inflammation and carcinogenesis mostly in the distal mouse colon, resembling left-sided colon and rectal cancers in humans [42]. All wildtype (wt) and *Xiap*^*IEC-KO*^ mice developed tumors in the distal colon 62 days after the 2% AOM/DSS treatment protocol. Conventional histopathology assessment of mouse tumor sections by Haematoxylin/Eosin (H&E) staining showed that the majority of lesions represented adenomas with few lesions progressing to adenocarcinomas (Fig. 2B). *Xiap*^*IEC-KO*^ mice showed significantly reduced afflicted intestinal area percentage compared to control mice (Fig. 2C). Although no changes in the total number of tumors were observed, larger tumors ( > 2 mm) were significantly less presented in *Xiap*^*IEC-KO*^ mice (Fig. 2C).

One of the central hurdles in exploring the role of elevated XIAP expression in cancer pathogenesis (initiation/progression) has been the lack of appropriate animal models for increased XIAP expression [14]. In order to model the elevated XIAP expression in XIAP-positive human CRC tumors, we generated a novel conditional *XIAP*-KI mice by targeting the Rosa26 locus. The expression of an N terminal HA-tagged mouse XIAP in this mouse model is prevented by a *LoxP*-flanked *STOP* cassette (*Rosa26*^*lSTOPlXiap*^) [43]. The transgenic mice express XIAP only after Cre-mediated excision of the *LoxP*-flanked *STOP* cassette. Thus, intercrossing of *Rosa26*^*lSTOPlXiap*^ with *Villin*^*Cre*^ generated mice with IEC-specific XIAP overexpression (*XIAP*^*IEC-KI*^) (Figure S2B).

*XIAP*^*IEC-KI*^ mice appeared normal and did not show any obvious phenotypic alteration (monitored for 6 months) of their intestinal tissues (Fig. 2A and Figure S2C) indicating that elevated XIAP expression in IEC does not promote malignant transformation in intestinal tissues. After applying the 2% AOM/DSS treatment protocol, *XIAP*^*IEC-KI*^ mice however showed a significant increase in tumor load as indicated by the total number of tumors and the percentage of afflicted area (Fig. 2D).

**Fig. 3:**
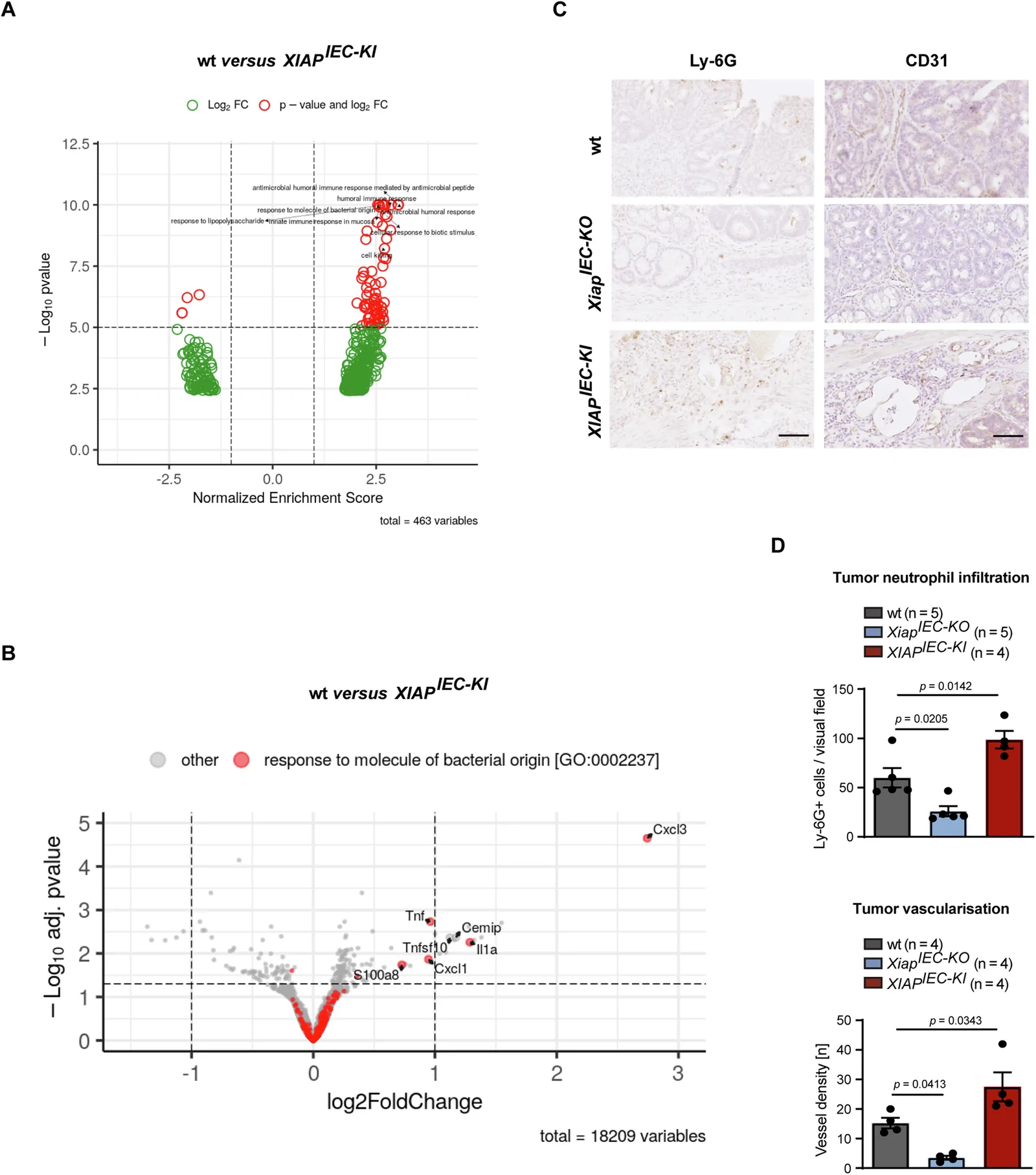
XIAP promotes inflammatory TME conditions in colitis-driven CRC in mice. **A** **GSEA plot** Gene Set Enrichment Analysis tests whether genes in a gene set tend to preferentially occur at the extreme ends of a sorted list of gene weights. Corresponding gene sets in different panels of the plot are identified by shared color. In all three panels, the x-axis represents positions in a sorted list of input genes. **(Top)** Each line corresponds to one gene set. At each x position ( = a given gene) a counter is incremented by this gene’s weight if the gene is in the gene set and decremented otherwise. **(Middle)** This panel shows a graph of gene position (x axis) versus gene weight (y axis). The log2FoldChange values of the differential expression analysis were used as weights. **(Bottom)** Each row in this panel represents one gene set as a barcode, indicating membership of a gene in the set by a bar at the gene’s x position. The names of the gene sets and the adjusted *p*-value of the observed enrichment are over-plotted over the barcodes. **B** **Volcano plot GSEA:** This plot relates the Normalized Enrichment Score (NES) of a gene set to the adjusted *p*-value of the enrichment. NES is a normalized version of the Enrichment Score (ES), which in turn is the maximum absolute deviation from zero observed in the Running Enrichment Score trace of the gene set. While the ES values of different gene sets depend on the gene set sizes, NES values of different gene sets can be meaningfully compared. Note that the default pvalueCutoff = 0.05 was in effect during the GSEA analysis. Hence non-significant gene sets do not show up in the volcano plot. **Volcano plot genes:** This plot relates the log2FoldChange of each gene to its adjusted *p*-value from the DESeq2 analysis. Genes belonging to the Gene Ontology (GO) term “response to molecule of bacterial origin” are marked in red, highlighting the fact that this entire large term tends to go up in the KI condition. **C** Representative immunohistochemistry images of CD31 and Ly-6G stainings of mouse adenomas of wt, *Xiap*^*IEC-KO*^, and *XIAP*^*IEC-KI*^ mice. Bar indicates 100 µm. **D** Quantification of tumor vascularization and neutrophil infiltration of 2% AOM/DSS induced murine colon adenomas of wt, *Xiap*^*IEC-KO*^, and *XIAP*^*IEC-KI*^ mice. Dots, individual mice. Lines, mean ± s.e.m. *P*-values by One-way ANOVA followed by Dunnett’s post-analysis.

Our data utilising *Xiap*^*IEC-KO*^ and *XIAP*^*IEC-KI*^ mice indicated that XIAP supports CRC progression. Analysis of cleaved caspase-3 (CASP3) in intestinal sections, however, did not reveal a dramatic change in IEC apoptosis upon *Xiap*^*IEC-KO*^ or *XIAP*^*IEC-KI*^ (Fig. 2E, F). Independently, XIAP promotes gene expression, which can potentially interfere with tumor growth [20, 21]. Therefore, we performed a tumor bulk RNA sequencing on tumors derived from wt and *XIAP*^*IEC-KI*^ mice. These analyses identified increased expression of inflammatory genes clustered in the ‘response to molecule of bacterial origin’ gene ontology pathway in *XIAP*^*IEC-KI*^ mice (Fig. 3A and Figure S3A). Specifically, elevated expression of several genes involved in controlling CRC TME such as *Cxcl3* (C-X-C Motif Chemokine ligand 3), *Il1a* (interleukin 1 alpha)*, Cemip* (cell migration-inducing and hyaluronan-binding protein, also called colon cancer secreted protein 1 (CCSP1))*, Tnfsf10* (tumor necrosis factor (TNF) superfamily member 10 or TNF-related apoptosis-inducing ligand (TRAIL))*, Tnf*, and *Cxcl1* was identified in *XIAP*^*IEC-KI*^ mice (Fig. 3B and Tables 1, 2). In particular, C-X-C motif chemokine ligands (CXCL) have been frequently associated with CRC progression by controlling tumor cell proliferation and motility, tumor vascularisation, and immune infiltration. CXCL family members and their cognate receptors have been lately considered to serve as non-invasive biomarkers for disease diagnosis [44]. CXCL1-8 contain the N-terminal three amino acid residues Glu-Leu-Arg (the “ELR” motif) that enable them to specifically bind to CXC receptor 1 (CXCR1) and CXCR2. CXCL members that contain the ELR motif are potent neutrophil chemoattractant and also serve as angiogenic factors [45, 46]. Increasing evidence identified CXCLs as potent mediator of CRC progression by efficiently inducing tumor neutrophil infiltration [47, 48]. Increased expression of *Cxcl1* and *Cxcl3* in tumors of *XIAP*^*IEC-KI*^ mice was associated with increased Ly-6G-positive neutrophilic granulocytes infiltration and elevated CD31-positive tumor vasculature (Fig. 3C, D). Furthermore, elevated expression of angiopoetin-2 (*Angpt2*) in tumor bulks derived from *XIAP*^*IEC-KI*^ mice (Tables 1, 2) might also explain the increased micro-vessel density (MVD) observed in these tumors (Fig. 3C, D). In contrast to neutrophils, aberrant tumor infiltration by T- or B-cells was not evident (Figure S3B). Together, these observations indicate that XIAP promotes inflammatory microenvironment, in particular, by inducing chemokine production which leads to the infiltration of tumors by neutrophils.

**Table 1:** Top 30 upregulated genes.

gene_name	gene_id	log2FoldChange	pvalue	padj	description
AC121792.4	ENSMUSG00000119774	21.43632874	2.6949E-13	4.54913E-09	NULL
Gm43302	ENSMUSG00000079362	21.21728437	4.70219E-13	4.54913E-09	predicted gene 43302 [Source:MGI Symbol;Acc:MGI:5663439]
Cxcl3	ENSMUSG00000029379	2.744156746	5.9565E-09	2.30505E-05	chemokine (C-X-C motif) ligand 3 [Source:MGI Symbol;Acc:MGI:3037818]
Mab21l3	ENSMUSG00000044313	1.54877468	1.24995E-06	0.002015437	mab-21-like 3 [Source:MGI Symbol;Acc:MGI:2446273]
Gm43042	ENSMUSG00000106634	1.382845024	5.41528E-06	0.004365846	predicted gene 43042 [Source:MGI Symbol;Acc:MGI:5663179]
Gm37347	ENSMUSG00000102975	1.327026746	8.51844E-06	0.004994644	predicted gene, 37347 [Source:MGI Symbol;Acc:MGI:5610575]
Il1a	ENSMUSG00000027399	1.287963424	1.0509E-05	0.005648079	interleukin 1 alpha [Source:MGI Symbol;Acc:MGI:96542]
Gm26670	ENSMUSG00000097416	1.277772056	1.89686E-06	0.002479345	predicted gene, 26670 [Source:MGI Symbol;Acc:MGI:5477164]
Cemip	ENSMUSG00000052353	1.172404325	6.58187E-06	0.004391471	cell migration inducing protein, hyaluronan binding [Source:MGI Symbol;Acc:MGI:2443629]
Btbd17	ENSMUSG00000000202	1.160497107	7.96864E-06	0.004994644	BTB (POZ) domain containing 17 [Source:MGI Symbol;Acc:MGI:1919264]
Tnfsf10	ENSMUSG00000039304	1.122052825	6.2163E-06	0.004391471	tumor necrosis factor (ligand) superfamily, member 10 [Source:MGI Symbol;Acc:MGI:107414]
Plekhs1	ENSMUSG00000035818	1.091295354	2.50555E-05	0.009695962	pleckstrin homology domain containing, family S member 1 [Source:MGI Symbol;Acc:MGI:2443041]
Hp	ENSMUSG00000031722	1.040523992	2.91664E-05	0.01035216	haptoglobin [Source:MGI Symbol;Acc:MGI:96211]
Fos	ENSMUSG00000021250	1.000588523	3.95325E-06	0.003642446	FBJ osteosarcoma oncogene [Source:MGI Symbol;Acc:MGI:95574]
Tnf	ENSMUSG00000024401	0.961537372	9.754E-07	0.00190866	tumor necrosis factor [Source:MGI Symbol;Acc:MGI:104798]
Cxcl1	ENSMUSG00000029380	0.947657411	4.534E-05	0.013925243	chemokine (C-X-C motif) ligand 1 [Source:MGI Symbol;Acc:MGI:108068]
Clca4b	ENSMUSG00000074195	0.797936646	8.4107E-05	0.019607107	chloride channel accessory 4B [Source:MGI Symbol;Acc:MGI:2139790]
Il18rap	ENSMUSG00000026068	0.76056484	6.8467E-05	0.017849908	interleukin 18 receptor accessory protein [Source:MGI Symbol;Acc:MGI:1338888]
S100a8	ENSMUSG00000056054	0.730162364	7.302E-05	0.018590368	S100 calcium binding protein A8 (calgranulin A) [Source:MGI Symbol;Acc:MGI:88244]
Tgm5	ENSMUSG00000053675	0.716159418	9.15939E-05	0.019690612	transglutaminase 5 [Source:MGI Symbol;Acc:MGI:1921426]
H2ac13	ENSMUSG00000071516	0.640058424	1.50571E-05	0.007666856	H2A clustered histone 13 [Source:MGI Symbol;Acc:MGI:2448457]
Vtcn1	ENSMUSG00000051076	0.58335743	0.000110421	0.021365443	V-set domain containing T cell activation inhibitor 1 [Source:MGI Symbol;Acc:MGI:3039619]
Angpt2	ENSMUSG00000031465	0.526175973	0.00019718	0.027290619	angiopoietin 2 [Source:MGI Symbol;Acc:MGI:1202890]
Retnlg	ENSMUSG00000022651	0.499590204	7.70908E-05	0.01864537	resistin like gamma [Source:MGI Symbol;Acc:MGI:2667763]
H2ac11	ENSMUSG00000069301	0.488210543	3.22148E-06	0.003167246	H2A clustered histone 11 [Source:MGI Symbol;Acc:MGI:2448293]
Phlda2	ENSMUSG00000010760	0.483403026	4.79572E-05	0.014275752	pleckstrin homology like domain, family A, member 2 [Source:MGI Symbol;Acc:MGI:1202307]
Egr1	ENSMUSG00000038418	0.473239008	0.000284366	0.03275112	early growth response 1 [Source:MGI Symbol;Acc:MGI:95295]
Vsig1	ENSMUSG00000031430	0.470298269	7.5242E-05	0.01864537	V-set and immunoglobulin domain containing 1 [Source:MGI Symbol;Acc:MGI:1926039]
5730507C01Rik	ENSMUSG00000073197	0.460143222	0.000275146	0.032362817	RIKEN cDNA 5730507C01 gene [Source:MGI Symbol;Acc:MGI:1917882]
Lipt2	ENSMUSG00000030725	0.439283889	6.76719E-05	0.017849908	lipoyl(octanoyl) transferase 2 (putative) [Source:MGI Symbol;Acc:MGI:1914414]

The table includes the to 30 upregulated genes in the tumors of *XIAP*^*IEC-KI*^ mice compared to wt mice.

**Table 2:** Top 30 downregulated genes.

gene_name	gene_id	log2FoldChange	pvalue	padj	description
Snhg11	ENSMUSG00000044349	−1.613157259	2.71594E-11	1.75169E-07	small nucleolar RNA host gene 11 [Source:MGI Symbol;Acc:MGI:2441845]
Hmgcs2	ENSMUSG00000027875	−1.363438652	1.73916E-06	0.002479345	3-hydroxy-3-methylglutaryl-Coenzyme A synthase 2 [Source:MGI Symbol;Acc:MGI:101939]
Zbtb16	ENSMUSG00000066687	−1.330189664	8.38399E-06	0.004994644	zinc finger and BTB domain containing 16 [Source:MGI Symbol;Acc:MGI:103222]
Srrm3	ENSMUSG00000039860	−1.294883145	9.25537E-06	0.005116631	serine/arginine repetitive matrix 3 [Source:MGI Symbol;Acc:MGI:1920309]
Gm43189	ENSMUSG00000105909	−1.217555483	9.24249E-06	0.005116631	predicted gene 43189 [Source:MGI Symbol;Acc:MGI:5663326]
Adam11	ENSMUSG00000020926	−1.12427159	6.47858E-06	0.004391471	a disintegrin and metallopeptidase domain 11 [Source:MGI Symbol;Acc:MGI:1098667]
Rgs11	ENSMUSG00000024186	−1.066207584	3.27381E-06	0.003167246	regulator of G-protein signaling 11 [Source:MGI Symbol;Acc:MGI:1354739]
Phf24	ENSMUSG00000036062	−0.930339465	1.03522E-06	0.00190866	PHD finger protein 24 [Source:MGI Symbol;Acc:MGI:2140712]
Tle2	ENSMUSG00000034771	−0.907888281	2.7983E-06	0.003008016	transducin-like enhancer of split 2 [Source:MGI Symbol;Acc:MGI:104635]
Gm37121	ENSMUSG00000103032	−0.88969132	2.71858E-06	0.003008016	predicted gene, 37121 [Source:MGI Symbol;Acc:MGI:5610349]
Gm35339	ENSMUSG00000109179	−0.840624069	1.70402E-07	0.000412137	predicted gene, 35339 [Source:MGI Symbol;Acc:MGI:5594498]
Ablim2	ENSMUSG00000029095	−0.833420051	6.33118E-06	0.004391471	actin-binding LIM protein 2 [Source:MGI Symbol;Acc:MGI:2385758]
Sgip1	ENSMUSG00000028524	−0.821175104	2.05382E-05	0.008781521	SH3-domain GRB2-like (endophilin) interacting protein 1 [Source:MGI Symbol;Acc:MGI:1920344]
Gm37006	ENSMUSG00000103885	−0.816429889	5.09336E-05	0.014414887	predicted gene, 37006 [Source:MGI Symbol;Acc:MGI:5610234]
Igkv8-30	ENSMUSG00000076577	−0.716803781	9.04406E-05	0.019690612	immunoglobulin kappa chain variable 8-30 [Source:MGI Symbol;Acc:MGI:3642250]
2900027M19Rik	ENSMUSG00000109162	−0.69782404	9.37609E-05	0.019690612	RIKEN cDNA 2900027M19 gene [Source:MGI Symbol;Acc:MGI:1920130]
Gm42769	ENSMUSG00000105922	−0.687465876	8.52808E-05	0.019644021	predicted gene 42769 [Source:MGI Symbol;Acc:MGI:5662906]
Nnat	ENSMUSG00000067786	−0.6872135	0.000118149	0.022194794	neuronatin [Source:MGI Symbol;Acc:MGI:104716]
Hottip	ENSMUSG00000055408	−0.683797629	2.23798E-05	0.009021396	Hoxa distal transcript antisense RNA [Source:MGI Symbol;Acc:MGI:3642509]
2900092N22Rik	ENSMUSG00000104034	−0.669253787	0.000143681	0.024314526	RIKEN cDNA 2900092N22 gene [Source:MGI Symbol;Acc:MGI:1920357]
Acacb	ENSMUSG00000042010	−0.662448037	1.9682E-05	0.008655168	acetyl-Coenzyme A carboxylase beta [Source:MGI Symbol;Acc:MGI:2140940]
Fmn2	ENSMUSG00000028354	−0.656999494	0.000128683	0.022843081	formin 2 [Source:MGI Symbol;Acc:MGI:1859252]
Efna5	ENSMUSG00000048915	−0.652390719	1.4776E-05	0.007666856	ephrin A5 [Source:MGI Symbol;Acc:MGI:107444]
Mib2	ENSMUSG00000029060	−0.609468037	2.28248E-08	7.3606E-05	mindbomb E3 ubiquitin protein ligase 2 [Source:MGI Symbol;Acc:MGI:2679684]
Meg3	ENSMUSG00000021268	−0.580482497	0.00017632	0.026863071	maternally expressed 3 [Source:MGI Symbol;Acc:MGI:1202886]
D130043K22Rik	ENSMUSG00000006711	−0.569987877	0.000189078	0.026929656	RIKEN cDNA D130043K22 gene [Source:MGI Symbol;Acc:MGI:3036268]
Sdk2	ENSMUSG00000041592	−0.557056902	0.00015953	0.025939038	sidekick cell adhesion molecule 2 [Source:MGI Symbol;Acc:MGI:2443847]
Adgrb2	ENSMUSG00000028782	−0.530167387	0.000227179	0.029196935	adhesion G protein-coupled receptor B2 [Source:MGI Symbol;Acc:MGI:2451244]
Zfp862-ps	ENSMUSG00000107476	−0.525705704	1.26481E-09	6.1182E-06	zinc finger protein 862, pseudogene [Source:MGI Symbol;Acc:MGI:1889827]
Rgs6	ENSMUSG00000021219	−0.518020915	0.000180916	0.026929656	regulator of G-protein signaling 6 [Source:MGI Symbol;Acc:MGI:1354730]

The table includes the to 30 downregulated genes in the tumors of *XIAP*^*IEC-KI*^ mice compared to wt mice.

**Fig. 4:**
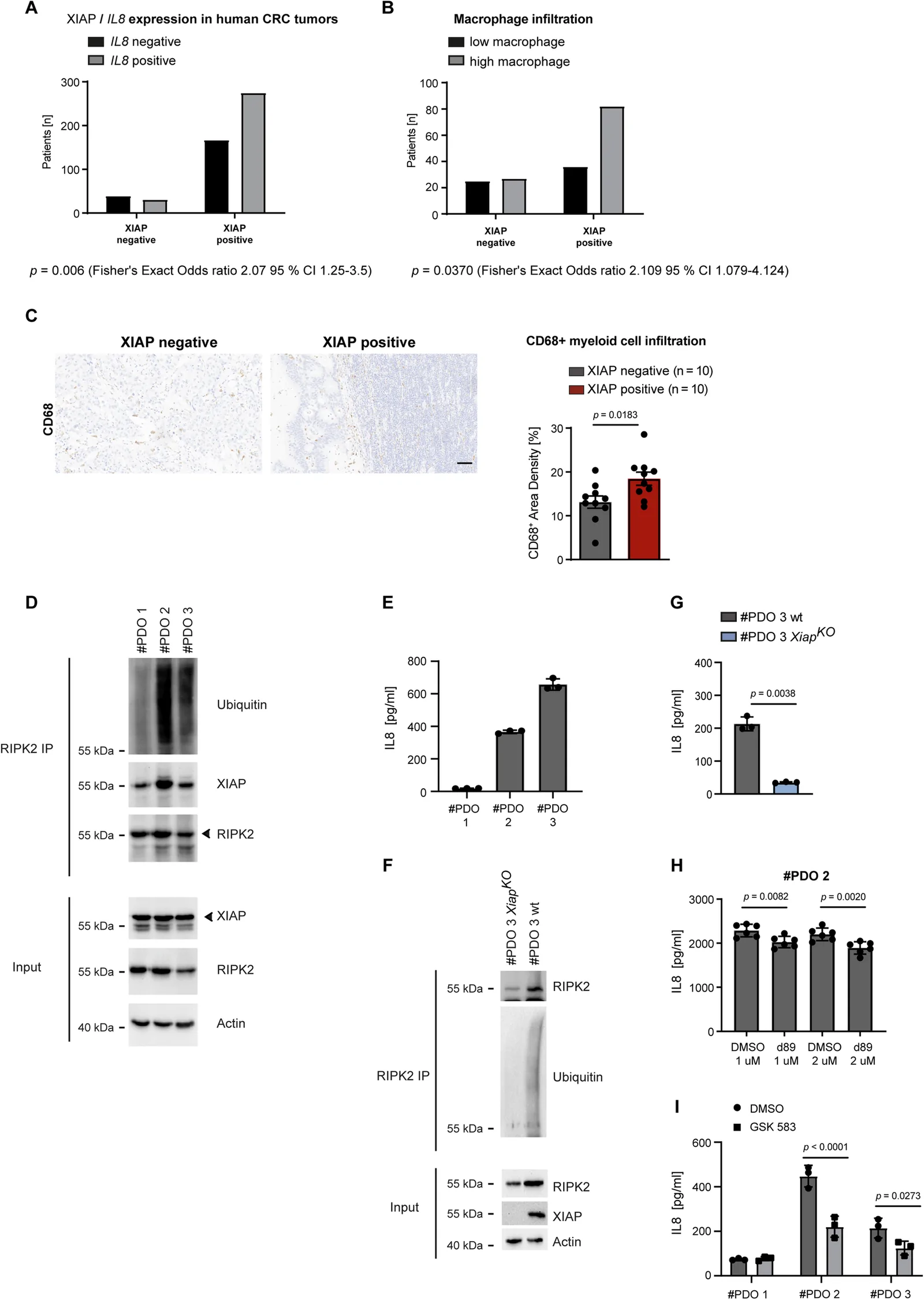
XIAP mediated shaping of inflammatory TME in CRC patients. **A** Grouped bar graphs of *IL8* abundance in human primary XIAP-negative and XIAP-positive colorectal tumors. **B** Grouped bar graphs of macrophage infiltration in human primary XIAP-negative (*n* = 52) and XIAP-positive (*n* = 118) colorectal tumors, identified by tissue microarray. *P*-values by Fisher’s exact test. **C** Representative immunohistochemistry images of CD68 staining of human primary colorectal tumors. Bar indicates 50 µm. Quantification of CD68^+^ macrophage infiltration of human primary colorectal tumors. Dots, individual patients. Lines, mean ± s.e.m. *P*-values by unpaired t-test. **D** Western blot analysis of lysates from patient-derived organoids (PDOs) (input) as indicated and RIPK2-IP Poly-ubiquitin. **E** IL8 measurement in the supernatant of the indicated organoids. **F** Western blot analysis of cell lysates from wt and *XIAP*^*KO*^ human organoids cells (input) and RIPK2-IP Poly-ubiquitin. **G** IL8 measurement in the supernatant of wt and *XIAP*^*KO*^ human organoids cells. Dots, individual biological replicates. Lines, mean ± SD. *P*-values by unpaired t-test. **H** IL8 measurement in the supernatant of the indicated organoid treated with d89 (BIR2 antagonist) or DMSO as a negative control. Dots, individual biological replicates. Lines, mean ± SD. *P*-values by ANOVA followed by Šídák post-analysis. **I** Representative IL-8 measurement in the supernatant of the indicated organoid treated with 2 uM GSK583 (RIPK2 kinase inhibitor) or DMSO as a negative control. Dots, individual technical replicates. Lines, mean ± SD. *P*-values by ANOVA followed by Šídák post-analysis.

In line with the data obtained in the CRC mouse model, the analysis of tissue microarrays of human CRC patient samples (*n* = 52 XIAP negative and *n* = 118 XIAP positive patients) showed that XIAP expression in human CRC was associated with an increased *CXCL8* (interleukin8; IL8) expression (Fig. 4A and Figure S4A) which is known to promote CRC progression and is associated with shorter overall survival and progression-free survival [49, 50]. For further verification of the role of XIAP in tumor immune infiltration, immune cell analysis in whole-mount sections derived from primary tumors of 10 XIAP-negative and 10 XIAP-positive patients was carried out. These data indicated that XIAP-positive patients have a significantly higher infiltration of CD68-positive myeloid cells (e.g. macrophages and neutrophils) (Fig. 4B-C).

Previous studies showed that XIAP-induced inflammation is normally mediated by ubiquitylation of RIPK2 which is followed by NFκB activity and expression of CXCL family members, such as IL8 [23, 28–30, 51]. In order to investigate the role of RIPK2 ubiquitylation in human CRC, we utilised CRC patient-derived organoids (PDO) and performed RIPK2 immunoprecipitation for further investigations [52]. Ubiquitylation of RIPK2 was only evident in two out of three independent PDOs (Fig. 4D), despite relatively similar XIAP expression levels between samples and interaction with RIPK2 which was further confirmed by XIAP immunoprecipitation (Figure S4D). Interestingly, RIPK2 ubiquitylation correlated with the production/secretion of IL8 from two CRC organoids (Fig. 4E). To more specifically analyse the involvement of XIAP in IL8 secretion, *XIAP* was knocked out in the high IL8-secreting organoid #PDO 3 using CRISPR Cas9. Organoids lacking XIAP failed to ubiquitylate RIPK2 and did not produce IL8 (Fig. 4F, G). These observations were further complemented by using a novel XIAP targeting compound XB2d89 (against human XIAP) [52]. The XIAP antagonist could reduce IL8 secretion in cultured human CRC organoids (Fig. 4H), further supporting the role of XIAP in chemokine secretion by mouse and human CRC. Additionally, RIPK2 kinase inhibition could also reduce IL8 secretion in cultured human CRC organoids (Fig. 4I). Expression of XIAP and RIPK2 was similar in all tested human CRC organoids (Figure S4E). Collectively, our data provide a rational to pharmaceutically target XIAP in CRC in order to dampen the tumor-supportive immune inflammatory microenvironment. To address the therapeutic value of XIAP, we decided to use a novel XIAP targeting compound XB2m54 [52] in the mouse AOM/DSS model. Mice were treated with XB2m54 every 2 days 29 days after initiating the protocol and tumors were harvested after 62 days (Figure S4B). Unfortunately, frequent exposure of mice to XB2m54 caused weight loss and treatments had to be stopped for some mice before we could collect statistically relevant data on tumor progression. Nevertheless, histological analysis of adenomas of these mice revealed a significant reduction in tumor vascular density upon XB2m54 treatment (Fig S4C), which resembles the data obtained in *Xiap*^*IEC-KO*^ mice and human tumor samples with low XIAP expression.

## Discussion

In the present study, we defined XIAP as a driver of CRC progression by dictating CRC-associated inflammation. Previous studies have already reported associations between XIAP expression and clinical outcomes in CRC [15]. However, these studies were primarily observational, based on retrospective analyses of human tissue samples, and generally interpreted XIAP in CRC in the context of its established anti-apoptotic function. Our study extends these observations by providing genetic, mechanistic, therapeutic, and in vivo evidence that XIAP has a previously unrecognized pro-inflammatory function that promotes CRC progression. The data provided in our study suggest the use of novel XIAP-targeting strategies as a valuable tool to treat inflammation-mediated cancer such as CRC, by specifically disrupting the pro-inflammatory signalling arm of XIAP involving RIPK2 [52].

Importantly, XIAP has been characterised as a potent caspase inhibitor rendering resistance to apoptosis [8]. However, in contrast to these findings, XIAP expression in some tumors did not interfere with the apoptotic propensity of tumor cells. Furthermore, additional biochemical evidence has revealed that XIAP has several cellular functions other than the regulation of apoptosis. Subsequently, the appropriateness of its name “X-linked inhibitor of apoptosis” has been called into question, as it becomes increasingly clear that this does not reflect the true nature of XIAP’s biological activities [14, 53]. The lack of appropriate animal models for conditional XIAP overexpression to model tumor tissues with elevated XIAP expression has been considered as the missing link to address the (patho-) physiological roles of XIAP in cancer [14]. A major strength of our study is therefore the establishment of two previously undescribed mouse lines and their corresponding controls, allowing us to induce colorectal epithelial-specific XIAP loss- and gain-of-function in the context of tumor development. Using these models, we demonstrate that XIAP promotes CRC progression in vivo and identify a distinct pro-inflammatory pathway involving RIPK2 and increased neutrophil infiltration of the tumors. To our knowledge, this provides the first genetic and physiological evidence linking epithelial XIAP to tumor-promoting inflammation and CRC progression in vivo.

Using a novel genetically engineered mouse model together with primary CRC organoids, we demonstrate that XIAP promotes inflammatory signalling through RIPK2 and consequently drives IL8 production and neutrophil infiltration into colorectal tumors. Importantly, the XIAP–RIPK2 inflammatory axis has previously been characterised predominantly in the context of bacterial infection [23, 28, 30]. We recently demonstrated that this inflammatory function of XIAP can also operate in human melanoma [51] and the present study extends this concept to CRC and establishes its functional relevance for inflammatory tumor progression. In line with these observations, accumulating recent evidence suggested RIPK2 as a potential prognostic factor for CRC [54, 55]. Our data involving inhibitors of XIAP or RIPK2 further provide convergent evidence that the XIAP–RIPK2 inflammatory axis is not merely associated with CRC progression but represents a functionally relevant pathway that may potentially be targetable in therapeutic protocols.

Our data suggests that XIAP-driven angiogenesis and inflammation constitute a critical driver of tumor progression, particularly in left-sided CRC. While right-sided tumors are often governed by immune-dominant pathways (MSI-High), we hypothesize that left-sided tumors appear to be ‘addicted’ to XIAP-mediated pro-angiogenic and pro-inflammatory signaling to overcome the constraints of their immunological ‘cold’ niche. This functional dependency on XIAP-driven immune cell recruitment and neovascularization provides a mechanistic rationale for why XIAP expression is a strong prognostic factor in left-sided colon cancer.

Taken together, XIAP has historically been studied predominantly as an anti-apoptotic protein and as a potential determinant or biomarker of chemoresistance, although the clinical literature on this association has produced conflicting results [14]. Our findings broaden this established view by demonstrating that XIAP can promote tumor progression independently of its canonical role in suppressing apoptosis, through activation of a pro-inflammatory RIPK2-dependent pathway. Thus, rather than simply supporting the existing paradigm that XIAP promotes cancer by preventing cell death, our data identify an additional, mechanistically distinct function of XIAP in regulating the TME and promoting CRC progression.

## Materials & Methods

### Patients

The tissue microarray (TMA) was constructed consisting of 558 tumor samples from patients with CRC (primary tumor samples, lymph node metastases and solid organ metastases) who had been treated between 1999 and 2014 at the Department of General, Visceral and Cancer Surgery, University of Cologne. 181 patients with primary colorectal tumors were eligible for XIAP analysis. Construction of the TMA was performed as previously described [56]. In brief, tissue cylinders with a diameter of 1.2 mm each were punched from selected tumor tissue blocks using a self-constructed semi-automated precision instrument and embedded in empty recipient paraffin blocks. 4 μm sections of the resulting TMA blocks were transferred to an adhesive coated slide system (Instrumedics Inc., Hackensack, NJ) for immunohistochemistry (IHC).

All procedures performed in studies involving human participants were in accordance with the ethical standards of the institutional research committee and with the 1964 Helsinki declaration and its later amendments or comparable ethical standards.

### Mice

All mouse studies were performed with approval by local government authorities (LAVE, NRW, Germany) in accordance with the German animal protection law with identification number 84-02.04.2017.A166. Animals were housed in the animal care facility of the University of Cologne under standard pathogen-free conditions with a 12 h light/dark schedule and provided with food and water ad libitum, temperature was between 20 and 24 °C and relative humidity between 45 and 65 rH.

Xiap ^fl/fl^ mice were inter-crossed with *Villin*^*Cre*^ mice to generate *Xiap*^*IEC-KO*^ mice and their control littermates (wt). To generate a mouse line that specifically overexpresses XIAP in intestinal epithelial cells (*XIAP*^*IEC-KI*^*)*, we generated the *Rosa26*^*LSL.HA-XIAP.IRES.GFP*^ mouse line, where expression of an N terminal HA-tagged mouse XIAP and downstream expression of GFP was prevented by a loxP flanked *STOP* cassette, and inter-crossed them with *Villin*^*Cre*^ mice (Vilin-Cre^Tg/wt^ *Rosa26*^*LSL.HA-XIAP.IRES.GFP/wt*^ Vilin-Cre^wt/wt^ *Rosa26*^*LSL.HA-XIAP.IRES.GFP/wt*^ were used as a control.

### Induction of mouse colorectal tumors in AOM/DSS model and antagonist treatment

A 8-week-old mice were i.p. injected with 10 mg/kg body weight AOM (A5486, Sigma-Aldrich) at day 1 of the 2% AOM/DSS protocol (Figure S2C). The animals were exposed to three repetitive cycles of 2% DSS (MW  =  36,000–50,000, 0216011080, MP Biomedicals) in the drinking water for 5 days from day 5−10, day 24–29 and day 43–48 (DSS water was refreshed every second day) to induce colitis. On day 62, animals were sacrificed by cervical dislocation at 17 weeks of age for tumor analysis. Mice that lost more than 20% of their body weight during the colitis phases were killed. At day 62, tumors were counted in a blinded fashion by number, size ( < 2 mm, > 2 mm) and percentage of the afflicted area of the distal colon.

To inhibit XIAP, mice were additionally treated with the XIAP inhibitor XB2m54 (Genentech, USA) by oral gavage every second day from day 29 until day 61 (100 mg/kg XB2m54 in 100 μl PBS or 100 μl PBS (Vehicle)).

### Quantitative PCR (qRT-PCR)

RNA isolation and qRT-PCR was done as described in Daoud et al. [51]. In short, RNA was isolated using the RNeasy Kit (Qiagen) followed by reverse transcription using the RevertAid Premium First Strand cDNA Synthesis Kit (Thermo Fisher) according to manufacturers’ instructions. qRT-PCR was run on a Multicolor Real-Time PCR Detection System (iQTM5, BIO-RAD) using the LightCycler® SYBR-Green I Mix (Roche Applied Sciences). Primers used: *Actb* fw: CTGAGAGGGAAATCGTGCGT, rev: AACCGCTCGTTGCCAATAGT; *Xiap* fw: TGGCAGAATATGAAGCACGGA, rev: CATGCTGTTCCCAAGGGTCT; *cIap1* fw: TGTGGCCTGATGTTGGATAAC, rev: GGTGACGAATGTGCAAATCTACT; *cIap2* fw: TGACGTGTGTGACACCAATG, rev: TGCTGCAGTGTTTCCTTTTG; human *Actb* fw: GGAGGAGCTGGAAGCAGCC, rev: GCTGTGCTACGTCGCCCTG; *Ripk2* fw: AGGGCCAGTATCAAGCACGA, rev: TCATACGTGCTCGGTGAGGT; *Xiap* fw: TGGGACATGGATATACTCAGTTAACAA, rev: GTTAGCCCTCCTCCACAGTGA. Data were analysed as previously described [57]. *Actb* was used as a housekeeping gene.

### Immunohistochemistry

Murine tissues were fixed in Roti-HistoFix 4% (Carl Roth), embedded in paraffin and cut in 5 μm sections. After rehydration and proteinase K treatment or heat-induced antigen retrieval in 10 mM citrate buffer, sections were stained for Ki67 (#ab16667, Abcam), cleaved Casp3 (#9661, Cell Signaling), F4/80 (#MOF4PU (V500), Immunostep), Ly-6G (#551459, BD Pharmingen), and CD31 (#550274, BD Pharmingen) as indicated. Goat Anti-Rabbit Biotin-Labeled (#NEF813001EA, Perkin Elmer) and Biotin-SP-AffiniPure goat anti-rat IgG (#112-065-003, Jackson Immunoresearch) were used as secondary antibodies. Staining was visualised using the ABC Kit Vectastain Elite (Vector Laboratories) and the DAB staining Kit (Dako) and counterstained with Hematoxylin (Carl Roth). The incubation time of the substrate for DAB staining was equal for all tissue sections. Tissue sections were also stained with H&E. The stained sections were scanned with the S360 Slidescanner (Hamamatsu) and analysed using the imaging software NDP.view2 2.9.22 (Hamamatsu) and ImageJ.

Expression of XIAP in human tumor sections was detected on TMA slides using a polyclonal rabbit anti-XIAP antibody (abcam, ab21278) on Leica BOND-MAX stainer (Leica Biosystems, Germany) according to the manufacturers’ protocol. CD68 was stained on standard whole mount sections using a monoclonal Mouse Anti-Human CD68 (Clone PG-M1, Agilent Dako, Germany) on a Leica Bond-Max stainer as well. Image acquisition, analysis and quantification was performed.

### Generation and culturing of patient-derived organoids (PDOs)

PDOs were obtained from surgically removed tumors after dissociation into single cells. Tumors were cut into small pieces and enzymatically digested at 4 °C using the tumor dissociation kit (Miltenyi, #130-095-929) following manufacturer’s instructions. 500 µl of enzyme mix were added to the tumor fragments in 5 ml of DMEM and placed in a gentleMACS C Tube (Miltenyi, #130-096-334). The gentleMACS Octo Dissociator with heaters (Miltenyi, #130-096-427) was used to dissociate the fragments using the default programs 37C_h_TDK_1. After that, single cells were seeded in 75% basement membrane extract (BME) and cultured in 50% Advanced DMEM/F12 (Gibco, #12634028) (supplemented with 1% L-Glutamine, 1% HEPES and 1% penicillin-streptomycin), 50% WRN conditioned media with supplements. The media was changed every other day. PDOs were split once a week using TrypLE (Invitrogen, #12605010).

### Generation of XIAP knockouts PDOs

PDOs were collected in a falcon, spun and the supernatant as well as the BME was discarded. The cell pellet was dissociated with TryplE, washed once with PBS and resuspended in 500 μl of media with 10 μg/ml of Polybrene (Sigma, #TR-1003). Two separate lentivirus transductions were performed. First, virus supernatant containing lentiviral particles encoding for the S. pyogenes Cas9 (a derivate of the lenti CRISPR v2 vector; lentiCRISPR v2 was a gift from Feng Zhang (Addgene plasmid # 52961)) were added to the organoid suspension and the mix was spun down at 32 °C for 30 minutes and incubated at 37 °C for 4 h. Transduced PDOs were plated in 70% BME. A total of 3−6 days after transduction eGFP+ single clones were isolated by FACS and picking single clones. After expansion of the organoids, a second transduction was performed in a similar manner with a lentivirus encoding for a single guide RNA targeting human *XIAP*. This time double positive cells for eGFP and mCherry were isolated and expanded. The knock-out was verified via Western blotting.

### Enzyme-linked immunosorbent assay (ELISA)

Human organoids were seeded on a 6-well plate. Medium was changed and collected after 24 h. Additionally, cells were treated with GSK583 (MedChem Express, #HY-100339), XB2d89 (d89) (Genentech, USA) or DMSO for 24 h and medium was changed and collected after 24 h. Analysis of secreted IL8 was performed using human CXCL8/IL8 DuoSet ELISA (R&D Systems) according to the manufacturer´s instructions.

### IL8 analysis - mRNA-in-situ (RNA-Scope)

The RNAscope assay was performed according to manufacturer’s instructions [58].

In brief, paraffin-embedded TMA blocks were cut in 5 μm sections, pretreated according to extended protocol (30 min for pretreatment 2 and 3), digested and hybridized at 40 °C in the HybEZ oven with human *IL8* mRNA probe provided by Advanced Cell Diagnostics Europe. Incubation time with Hematoxylin was 10 seconds. Target expression was compared to both negative (dapB) and positive (PPIB) controls. Scoring of signals was done as recommended by the manufacturer with no staining or less than one molecule per 10 cells = score 0, 1-3 dots/cell = score 1, 4−9 dots/cell = score 2, 10−15 dots/cell = score 3 and > 15 dots/cell =  score 4. DapB score was 0 and PPIB score was 2. Positivity was defined as a score > 0.

Strategy of evaluation: In addition to the extent of mRNA signal within individual cells, the number of *IL8* mRNA–expressing cells within the tissue was also taken into account. We assessed *IL8* mRNA expression in tissue sections by semiquantitative evaluation of the RNAscope signal across the entire population. First, all cases were reviewed comprehensively to capture the overall extent of IL8-positive tumor-associated cells within each specimen. Based on this global semiquantitative assessment, each tumor was assigned an individual IL8 expression score. To dichotomize the cohort, the median score of all evaluated tumors was used as the predefined cutoff. Tumors with scores above the median were classified as IL8-rich, whereas tumors with scores at or below the median were classified as IL8-poor. This median-based approach allowed cohort-internal stratification without applying external or biologically arbitrary threshold values.

### Immunoprecipitation (IP)

IP of endogenous ubiquitylated RIPK2 was done as described previously [51, 59]. In brief, cell pellet was resuspended in RIPA buffer (1% Triton, 150 mM NaCl, 50 mM Tris, 1% SDS, 0.5% deoxycholate) supplemented with N-ethylmaleimide (NEM) (Sigma-Aldrich) and 1× complete protease inhibitor cocktail (Roche) followed by 30-min incubation on ice and centrifugation for 20 min at 20,000 g at 4 °C. SDS concentration was diluted to 0.1% SDS prior to antibody incubation. Lysates were incubated for 1 h with antibody against RIPK2 (#4142, Cell Signaling) at 4 °C on a rotating wheel followed by overnight incubation with Protein A/G Plus agarose beads (sc-2003, Santa Cruz).

IP of endogenous XIAP was done by washing the cells chilled PBS followed by centrifugation at 700 *g* for 3 min. The cell pellet was resuspended in lysis buffer (Miltenyi Biotec) in addition to *N*-ethylmaleimide (NEM) (Sigma-Aldrich) and 1× complete protease inhibitor cocktail (Roche) followed by 30 min incubation on ice and centrifugation for 20 min at 20,000 *g* at 4 °C. Supernatant was collected and incubated first 1 h with a respective antibody against XIAP (#14334, Cell Signaling) at 4 °C on a rotating wheel followed by overnight incubation with Protein A/G Plus agarose beads (sc-2003, Santa Cruz).

Beads were washed 3× with PBST (PBS + 0.1% Tween 20). Protein concentration was performed with Pierce™ BCA Protein Assay Kit (Thermo Fisher Scientific). Proteins were separated by SDS-PAGE and transferred to a nitrocellulose membrane (Amersham Protean, GE). Proteins were stained with antibodies against XIAP (#610763, BD Biosciences), RIPK2 (#4142, Cell Signaling), Ubiquitin (#3936, Cell Signaling) and β-Actin (#sc-47778 HRP, Santa Cruz). Secondary antibodies included goat anti-rabbit IgG conjugated to horseradish peroxidase (HRP) (#7074, Cell Signaling) and goat anti-mouse IgG light chain HRP (#58802, Cell Signaling). Uncropped Western blots can be found in Supplementary Materials.

### Bioinformatic analysis

#### Differential expression

RNA-seq was performed with a directional protocol. Quality control, trimming, and alignment were performed using the nf-core RNA-seq pipeline (v3.6) [60]. Details of the software and dependencies for this pipeline can be found at the referenced DOI for the pipeline and: https://github.com/nf-core/rnaseq/blob/master/CITATIONS.md. The reference genome sequence and transcript annotation used were Mus musculus genome GRCm39 from Ensembl version 105.

nf-core commands:

## nextflow pull nf-core/rnaseq -r 3.6

##

## nextflow run nf-core/rnaseq \

## --input samplesheet.csv \

## --fasta ‘http://ftp.ensembl.org/pub/release-105/fasta/mus_musculus/dna/Mus_musculus.GRCm39.dna.primary_assembly.fa.gz’ \

## --gtf ftp://‘http://ftp.ensembl.org/pub/release-105/gtf/mus_musculus/Mus_musculus.GRCm39.105.gtf.gz’ \

## --gtf_count_type exon --gtf_group_features gene_id --gtf_extra_attributes gene_name \

## --save_reference \

## --aligner star_salmon \

## -profile singularity \

## --outdir. /results_RNA-seq \

## -c nf-conf.conf \

## -r 3.6 \

Differential expression analysis was performed in R version 4.1.2 (2021-11-01) (R Core Team 2021) with DESeq2 v1.34.0 [61] to make pairwise comparisons between groups. Log Fold Change shrinkage estimation was performed with ashr. Only genes with a minimum coverage of 10 reads in 6 or more samples from each pairwise comparison were considered as candidates to be differentially expressed. Genes were considered differentially expressed if they showed a *|log2(Fold Change)| *> *0.25* and were below an FDR of 0.05.

#### Functional enrichment

Genes with a minimum coverage of 10 reads in 6 or more samples from each pairwise comparison were included in functional enrichment analyses and considered as the ‘gene universe’ for over-representation-based analyses. Functional enrichment analysis was performed with clusterProfiler v4.2.2 [62]. Gene Set Enrichment Analysis (GSEA) [63] was performed using the fgsea v1.20.0 algorithm implemented in clusterProfiler. The GSA plot was drawn by a local modification of enrichplot::gseaplot2 v1.14.2, as re-exported by clusterProfiler.

## Supplementary information

Figure S1

Figure S2

Figure S3

Figure S4

Supplementray Figure Legends

Original Data

## Data Availability

Primary data are available from the corresponding author upon request.

## Code availability

The repository can be viewed and cloned from: https://github.com/CECAD-Bioinformatics-Facility-Projects/Role-of-XIAP-in-Colorectal-Cancer/
