# Supplementary material for: XIAP induces inflammation and promotes colorectal cancer progression
Source: Cell Death Dis. 2026 Nov 2;17(1):931. doi: 10.1038/s41419-026-09391-9 (PMC13728099; doi:10.1038/s41419-026-09391-9)

Fig 4

D

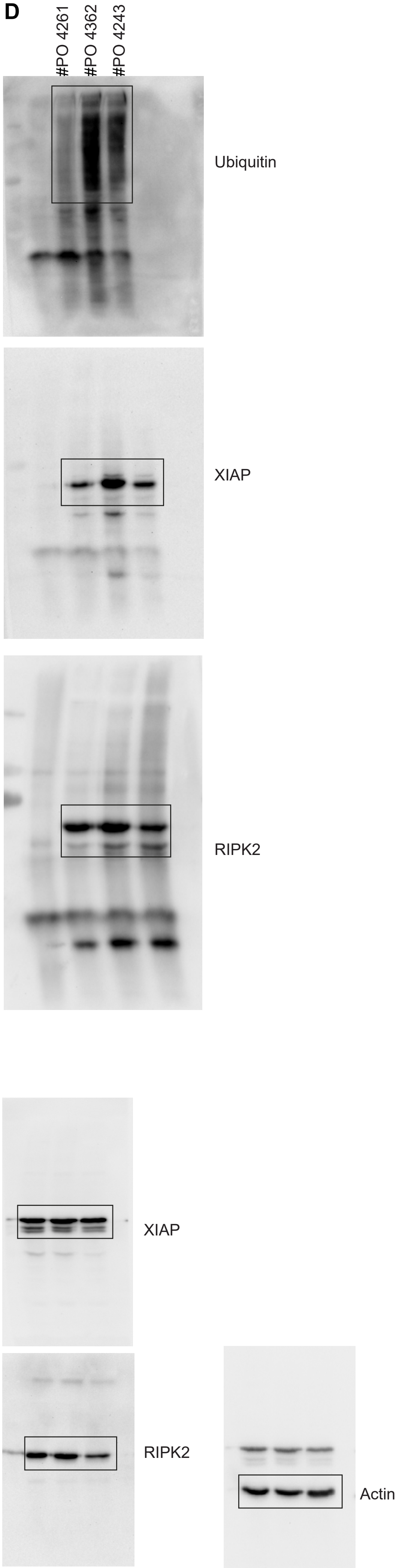

F

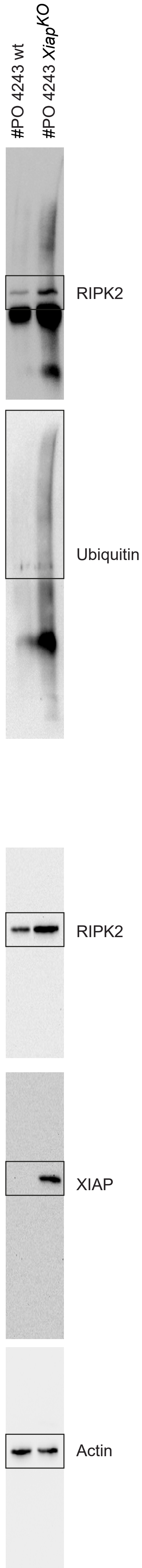

Fig. S4

D

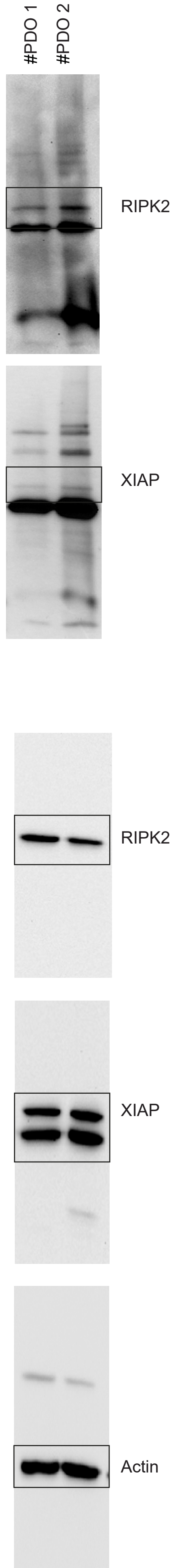

Supplement: Supplementary file 1 [file 41419_2026_9391_MOESM6_ESM.pdf]
