# Supplementary material for: XIAP induces inflammation and promotes colorectal cancer progression
Source: Cell Death Dis. 2026 Nov 2;17(1):931. doi: 10.1038/s41419-026-09391-9 (PMC13728099; doi:10.1038/s41419-026-09391-9)
Supplement: Supplementary file 2 [file 41419_2026_9391_MOESM5_ESM.docx]

Supplementary Figure Legends

**Fig S1 XIAP expression in primary resected CRC patients.**

**(A)** Kaplan-Meier curve showing overall survival of patients stratified for XIAP immune-score 0-3 (n = 181). **(B)** Table depicting basic clinical characteristics of patients included in the analysis. **(C)** Table showing results of multivariate analysis identifying XIAP as an independent predictor of overall survival.

**Fig S2 Establishment of colitis-driven CRC in mice with altered XIAP expression.**

**(A-B)** mRNA levels of *Xiap*, *cIap1*, and *cIap2* in tumor samples of (A) *Xiap^IEC-KO^* and (B) *XIAP^IEC-KI^* compared to wt by RT-PCR. Dots, individual mice. Lines, mean ± s.e.m. P-values by unpaired t-test. **(C)** Representative images of ileum sections of untreated wt (n = 3), *Xiap^IEC-KO^* (n = 2) and *XIAP^IEC-KI^* (n = 3) mice. Bars indicate 150 µm for H&E, and 100 µm for F4/80, Ly-6G, and cl. Caspase 3. **(D)** Scheme depicting the experimental schedule of the 2 % AOM/DSS protocol.

**Fig S3 XIAP promotes immune inflammatory signalling in mouse CRC.**

**(A)** Gene set enrichment analysis **(B)** Representative images of colon sections of untreated wt (n = 5), *Xiap^IEC-KO^* (n = 5) and *XIAP^IEC-KI^* (n = 4) mice. Bars indicate 100 µm.

**Fig S4 XIAP inhibitor Xb2m54 reduces tumor vascularization in colitis-driven CRC in mice.**

**(A)** Representative immunohistochemistry images of *IL8* staining of human primary colorectal tumors. Bar indicates 50 µm. **(B)** Scheme depicting the experimental schedule of the 2 % AOM/DSS protocol with additional treatment with the murine XIAP inhibitor Xb2m54. **(C)** Representative immunohistochemistry images (left) and quantification (right) of CD31 staining of 2 % AOM/DSS induced mouse adenomas of C57BL/6 wt mice treated with either Vehicle (PBS) or XB2m54 (100 mg/kg). Bar indicates 100 µm. Dots, individual mice. Lines, mean ± s.e.m. P-values by unpaired t-test. **(D)** Western blot analysis of lysates from patient-derived organoids (PDOs) (input) as indicated and XIAP-IP. **(E)** mRNA levels of *XIAP* and *RIPK2* in the indicated organoids treated with 2 uM GSK583 (RIPK2 kinase inhibitor) or DMSO as a negative control by RT-PCR. Dots, individual biological replicates. Lines, mean ± SD.
